# Simultaneous enhancement of T_1_ and T_2_ magnetic resonance imaging of liver tumor at respective low and high magnetic fields

**DOI:** 10.7150/thno.67155

**Published:** 2022-01-01

**Authors:** Huan Li, Zijuan Hai, Liwei Zou, Lele Zhang, Lulu Wang, Longsheng Wang, Gaolin Liang

**Affiliations:** 1 Department of Radiology, the Second Hospital of Anhui Medical University, Hefei, Anhui 230601, China.; 2 Key Laboratory of Structure and Functional Regulation of Hybrid Materials, Ministry of Education, Institutes of Physical Science and Information Technology, Anhui University, Hefei, Anhui 230601, China.; 3 State Key Laboratory of Bioelectronics, School of Biological Science and Medical Engineering, Southeast University, Nanjing, Jiangsu 210096, China.; 4 High Magnetic Field Laboratory, Hefei Institutes of Physical Science, Chinese Academy of Sciences, Hefei, Anhui 230031, China.

**Keywords:** γ-glutamyl transpeptidase, gadolinium nanoparticle, magnetic resonance imaging, liver tumor, tumor imaging

## Abstract

**Background**: Nowadays, magnetic resonance imaging (MRI) is routinely applied in clinical diagnosis. However, using one contrast agent (CA) to simultaneously enhance the T_1_ and T_2_ MR contrast at low and high magnetic fields respectively has not been reported.

**Methods**: Herein, we investigated the MR property of a γ-glutamyl transpeptidase (GGT)-instructed, intracellular formed gadolinium nanoparticle (DOTA-Gd-CBT-NP) at low and high magnetic fields.

**Results**: Experimental results showed that DOTA-Gd-CBT-NP possesses a low r_2_/r_1_ ratio 0.91 which enables it to enhance T_1_ MR imaging of liver tumor at 1.0 T, and a high r_2_/r_1_ ratio 11.8 which renders the nanoparticle to largely enhance T_2_ MR imaging of liver tumor at 9.4 T.

**Conclusion:** We expect that our GGT-responsive Gd-nanoparticle could be applied for simultaneous T_1_ and T_2_ MRI diagnosis of early liver cancer in clinic at respective low and high magnetic fields when the 9.4 T MR machine is clinically available in the future.

## Introduction

Magnetic resonance imaging (MRI) has turned to be one of the most widely used clinical diagnostic technologies due to its noninvasiveness, superb spatial resolution, and high contrast in soft tissues 1-3. However, the low sensitivity of MRI limits its ability to differentiate the pathological region from normal tissues. To further improve MRI contrast, approximately 35% of clinical MRI scans need exogenous contrast agents (CAs) 4,5. They are mainly divided into two types: (1) T_1_ CAs, such as paramagnetic gadolinium (Gd) complexes, primarily shorten the longitudinal relaxation time (T_1_) of protons and result in bright contrast; (2) T_2_ CAs, such as superparamagnetic iron oxide (SPIO) nanoparticles, mainly reduce the transverse relaxation time (T_2_) of protons and result in dark contrast 6,7. Generally, T_1_ MRI is advantageous in evaluating fat tissues or liquid-fixing concrete structures (e.g., joints), and T_2_ MRI is powerful in assessing water-rich tissues (or organs) or regional inflammations 8. The ratio between transverse and longitudinal relaxivities (i.e. r_2_/r_1_) of a CA is a defining parameter to predict its MRI type. In general, MRI CAs with small r_2_/r_1_ ratio (< 5) serve as T_1_ CAs and with large r_2_/r_1_ ratio (> 8) act as T_2_ CAs 9,10.

Up to now, varies of T_1_ CAs, T_2_ CAs, T_1_/T_2_ switchable CAs and T_1_-T_2_ dual modal CAs have been developed to increase MRI contrast at low or high magnetic field 11-19. However, using one CA to simultaneously enhance the T_1_ and T_2_ MR contrasts at low and high magnetic fields respectively has not been reported so far. Currently, small molecule Gd chelates, such as Gd-diethylenediaminepentaacetic acid (Gd-DTPA, Magnevist) and Gd-tetraazacyclododecanetetraacetic acid (Gd-DOTA, Dotarem), have been commonly employed as T_1_ CAs in clinical imaging. Nevertheless, these Gd chelates are small molecules and therefore their body circulation times are short and biodistributions are nonspecific 20-22. As a result, stimuli-responsive nanoscale CAs which could efficiently improve the specificity and sensitivity of MRI have attracted great attention. In previous works, we found that reduction-controlled self-assembled Gd-nanoparticle could been used as T_1_ CAs at low magnetic field (0.5, 1.5, 3.0 T) while enzyme-instructed self-assembled Gd-nanofiber (or Gd-nanoparticle) could enhance T_2_-weighted MR contrast at high magnetic field (9.4 T) 6,23,24. Therefore, we estimate that self-assembled Gd-nanoparticle might have the potency to simultaneously act as T_1_ and T_2_ CA at low and high magnetic fields respectively.

Liver cancer is one of the most common human malignancy. Although surgical (including liver resection and liver transplantation) and non-surgical (including transarterial chemoembolization, radiotherapy, and ablation) treatments have been applied in clinical practice, the 5-year survival rate of liver cancer patients is still less than 30% 25-27. Early precise diagnosis allows the patients to receive the treatment earlier and achieve better survival rates. During the occurrence of liver cancer, levels of some trace biomarkers in tissues and body fluids are abnormal. Accurate detection of these biomarkers can specifically distinguish the abnormal pathological processes, which is the key to realize early diagnosis of liver cancer 28. γ-glutamyl transpeptidase (GGT) is an enzyme characterized by it cell surface-binding property and plays important roles in various physiological processes. Up to date, numerous studies have reported that elevated serum GGT level is closely correlated with liver cancer patients 29,30. Hence, endogenous GGT is a promising biomarker for early diagnosis of liver cancer. Since MRI is commonly used in clinical liver cancer diagnosis and evaluation 31,32, it is of great importance to exploit new biomarker-responsive MRI CAs for early precise diagnosis of liver cancer.

**Figure 1:**
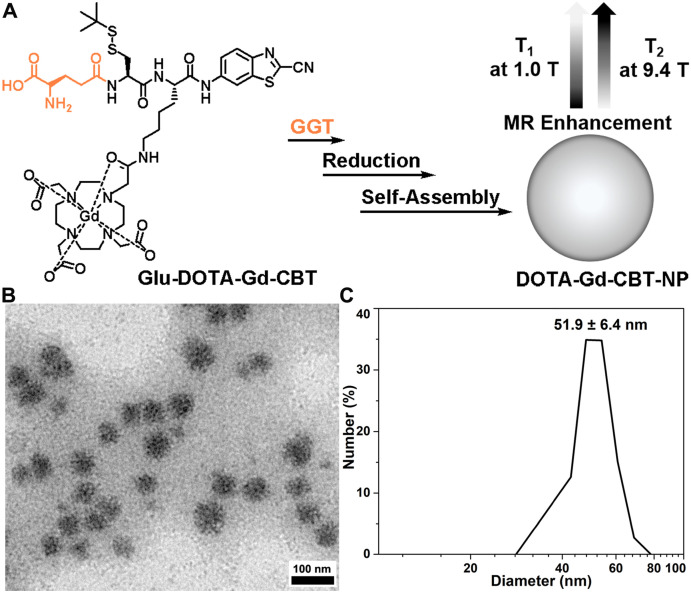
(A) Schematic illustration of GGT-guided formation of **DOTA-Gd-CBT-NP** to enhance T_1_ and T_2_ MR contrasts at low (1.0 T) and high (9.4 T) magnetic fields, respectively. (B) TEM image and DLS measurement (C) of **DOTA-Gd-CBT-NP** in PBS buffer.

Inspired by above studies, we recalled the “smart” Gd-based probe in our previous work which could intracellularly self-assemble into Gd-nanoparticle under the activation of GGT with T_2_ enhancement of subcutaneous tumor at 9.4 T 24. Generally, at increasing magnetic field strengths, r_1_ typically decreases while r_2_ is static or increases resulting in an increasing r_2_/r_1_ ratio. Specifically, for paramagnetic contrast agents based on Gd or manganese (Mn), the magnetization of the complexes increases linearly with magnetic field strength and this results in higher r_2_ values of the complexes at higher magnetic field 19,33-35. In this work, we intended to systematically investigate its MR property for liver tumor imaging at both low (1.0 T) and high (9.4 T) magnetic fields. As shown in Figure 1A, Glu-DOTA-Gd-CBT was consisted of three parts: a GGT-responsive substrate, motifs for 2-cyano-benzothiazole (CBT)-Cysteine (Cys) click reaction, and DOTA-Gd for MRI. Under the process of cell uptake, Glu-DOTA-Gd-CBT is cleaved by GGT at cell surface. Then after reduction by intracellular glutathione (GSH), it self-assembles into Gd-nanoparticle (i.e., DOTA-Gd-CBT-NP) via π-π stacking *in situ*. We found that DOTA-Gd-CBT-NP possesses a low r_2_/r_1_ ratio 0.91 at 1.0 T which enables it to enhance T_1_ MR imaging of liver tumor by 12.7% at low magnetic field and a high r_2_/r_1_ ratio 11.8 at 9.4 T which renders it to enhance T_2_ MR imaging of liver tumor by 19.7% at high magnetic field.

## Materials and Methods

**Materials and instruments**

**Figure 2:**
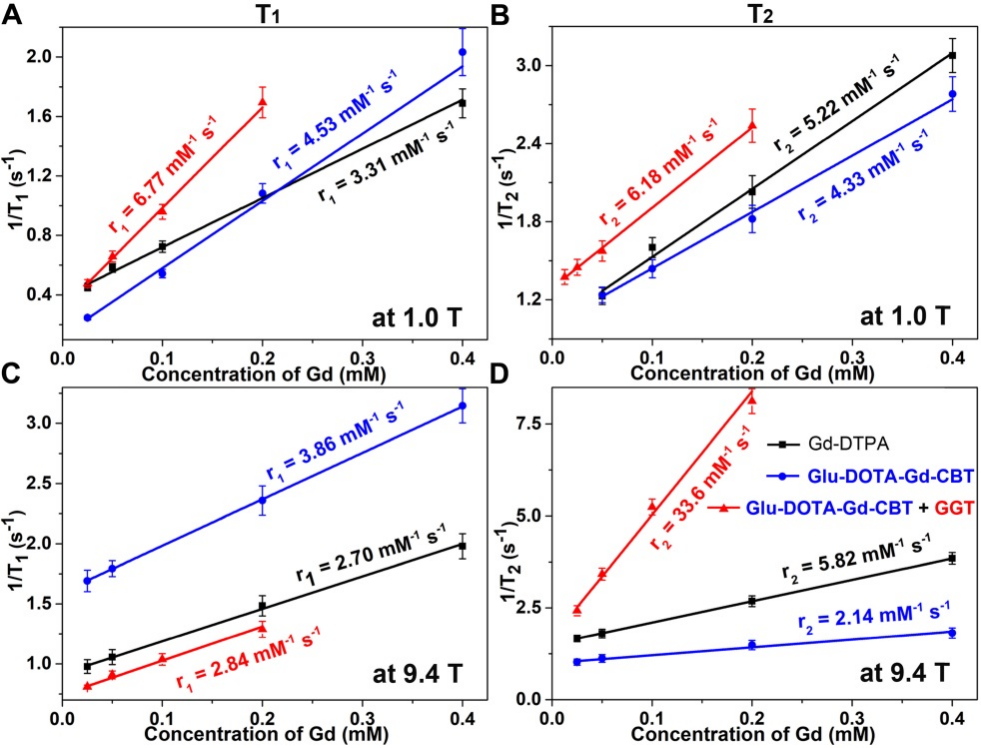
Longitudinal relaxation rates (1/T_1_) and transverse relaxation rates (1/T_2_) of Group **Glu-DOTA-Gd-CBT** + GGT (i.e., **DOTA-Gd-CBT-NP**), Group **Glu-DOTA-Gd-CBT**, and Group Gd-DTPA at low (1.0 T) or high (9.4 T) magnetic field.

**Figure 3:**
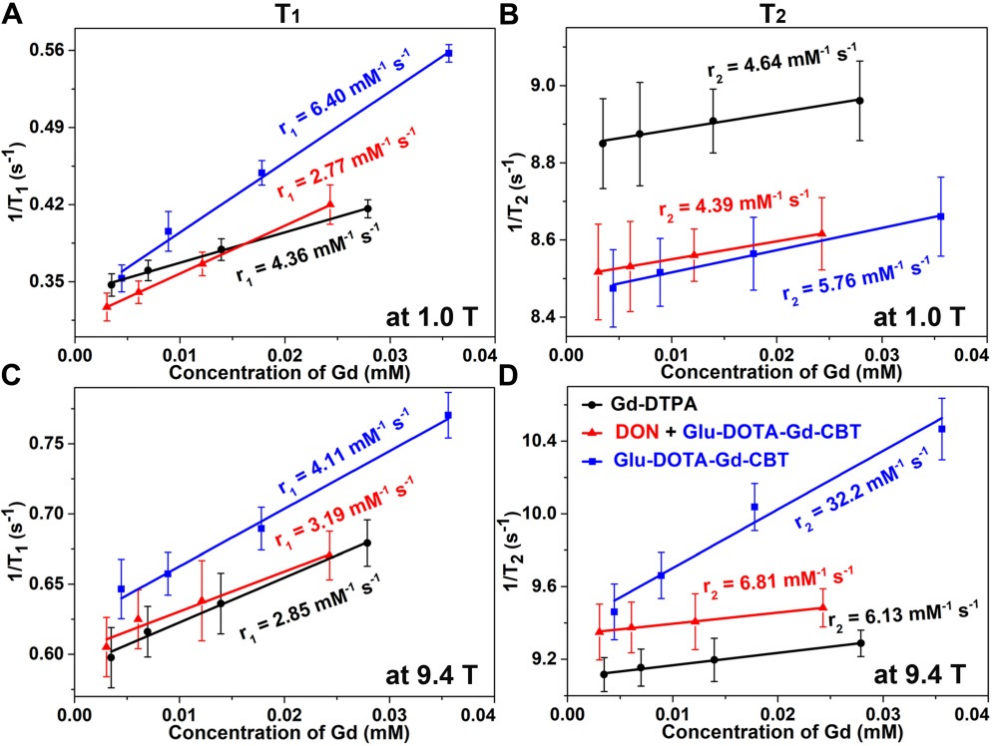
Longitudinal relaxation rates (1/T_1_) and transverse relaxation rates (1/T_2_) of HepG2 cells in Group **Glu-DOTA-Gd-CBT**, Group “DON + **Glu-DOTA-Gd-CBT**”, and Group Gd-DTPA at low (1.0 T) or high (9.4 T) magnetic field.

All the starting materials were obtained from Sigma, Adamas or GL Biochem. Commercially available reagents were used without further purification, unless noted otherwise. All chemicals were reagent grade or better. Transmission electron micrograph (TEM) images were obtained on a JEM‐2100F field emission transmission electron microscope operated at an acceleration voltage of 200 kV. MRI studies at low magnetic field were performed on a 1.0 T small animal MRI system (Bruker ICON). MRI studies at high magnetic field were performed on a 9.4 T/400 mm wide bore scanner (Agilent Technologies, Inc., Santa Clara, CA, USA) using a volume RF coil. The concentrations of Gd were determined with Inductively Coupled Plasma-Mass Spectrometry (ICP-MS) on the Perkin Elmer Optima 7300DV spectrometer. Each error bar in Figures 2 and 3 in the main text represents the standard deviation of three independent experiments.

### Cell experiment

HepG2 cells were divided into three groups: Group **Glu-DOTA-Gd-CBT** was the cells incubated with 200 μM **Glu-DOTA-Gd-CBT** for 4 h; Group “DON + **Glu-DOTA-Gd-CBT**” was the cells pretreated with 2 mM DON for 0.5 h then incubated with 200 μM **Glu-DOTA-Gd-CBT** for 4 h; Group Gd-DTPA was the cells incubated with 200 μM Gd-DTPA for 4 h. For MRI of living cells, HepG2 cells in three groups were diluted into four concentrations and resuspended in serum-free culture medium with 1 wt% agarose gel.

### Construction of liver tumor *in situ*

All animals received care according to the guidelines of the Care and Use of Laboratory Animals. The procedures were approved by the Anhui Medical University Animal Care and Use Committee (LLSC20210378). 6-8 weeks old (weighting about 18 g) male BALB/c nude mice were used for animal experiments under SPF environment. Human hepatoma cells marked with red fluorescent protein (HepG2-RFP) in logarithmic growth phase were subcutaneous inoculated to male BALB/c nude mice. Dissect the tumor when the HepG2-RFP subcutaneous mass grows to about 500 mm^3^ and then divided into 1 mm × 1 mm × 1 mm tumor tissue in the medium. The mice were anesthetized with isoflurane anesthesia. After the pain of the nude mice disappeared, a 1 cm transverse incision was made in the lower abdomen of the nude mice under the microscope of 8x surgery with the method of microsurgery, the liver was exposed after cut the skin and peritoneum, and the tumor tissue was transplanted into the liver. Then close the abdominal cavity with 5-0 surgical sutures. The whole operation process is completed in the super clean workbench. The experiment will be carried out when the estimated average volume of the tumor grows about 1cm × 1cm × 1cm by the *in vivo* fluorescence imaging system.

### *In vitro* and *in vivo* MRI experiments at 1.0 T

For *in vitro* MRI experiments, the longitudinal relaxation time (T_1_) were measured with a spin echo (SE) sequence using parameter of echo time (TE) = 9 ms, nineteen repetition times (TR) as follows: 20, 40, 60, 80, 100, 150, 200, 400, 600, 800, 1000, 1500, 2000, 4000, 6000, 8000, 10000, 15000, and 30000 ms, field of View (FOV) = 35 mm × 35 mm, matrix size = 256 × 256, and 3 slices with slice thickness = 4 mm. The transverse relaxation time (T_2_) were measured with a multi-echo spin echo sequence using parameter of TR = 2500 ms, eleven echo times (TE) ranging from 16 to 176 ms, field of View (FOV) = 35 mm × 35 mm, matrix size = 192 × 192, and 3 slices with slice thickness = 4 mm.

For *in vivo* MRI studies, T_1_-weighted MR images were acquired using fast spin echo sequence with following parameters: field of View (FOV) = 35 mm × 35 mm, matrix size = 256 × 256, slice thickness = 1 mm (10 slices, gap = 0), repetition time (TR) = 446 ms, echo spacing = 15 ms, effective echo time (TE) = 15 ms, 16 averages, rare factor = 2. T_2_-weighted MR images were acquired using fast spin echo sequence with following parameters: field of View (FOV) = 35 mm × 35 mm, matrix size = 192 × 192, slice thickness = 1 mm (10 slices, gap = 0), repetition time (TR) = 2905 ms, echo spacing = 28 ms; effective echo time (TE) = 84 ms, 6 averages, rare factor = 8.

### *In vitro* and *in vivo* MRI experiments at 9.4 T

For *in vitro* MRI experiments, the longitudinal relaxation time (T_1_) were measured with a rapid acquisition with relaxation enhance sequence using parameter of echo time (TE) = 16.18 ms, eight repetition times (TR) as follows: 843, 1117.378, 1141.050, 1835.969, 2342.620, 3050.310, 4233.124 and 9000 ms, field of View (FOV) = 32 mm × 32 mm, matrix size = 128 × 128, BW 50 kHz, and 5 slices with slice thickness = 1 mm. The transverse relaxation time (T_2_) were measured with a multi-echo spin echo sequence using parameter of TR = 5,000 ms, thirty echo times (TE) ranging from 12 to 360 ms, echo spacing = 12 ms, field of view (FOV) = 32 mm × 32 mm, matrix size = 128 × 128, BW 50 kHz, and 5 slices with slice thickness = 1 mm.

For *in vivo* MRI studies, the longitudinal relaxation time (T_1_) were measured using following parameters: field of view (FOV) = 25 mm × 25 mm; matrix size = 128 × 128; TE = 12 ms, echo spacing = 6 ms, rare factor = 4, T_1_ experiments = 6 and 15 slices, slice thickness = 1 mm, 1 average. Six inversion times (TI) were used varied from 550 to 1800 ms. The transverse relaxation time (T_2_) were measured with a multi-echo spin echo sequence using parameter of TR = 3000 ms, twenty echoes with echo times (TE) ranging from 8 to 160 ms, with field of View (FOV) = 25 mm × 25 mm and matrix size = 128 × 128, slice thickness = 1 mm (15 slices, gap = 0); echo averages = 1.

## Results and Discussion

### MR property of DOTA-Gd-CBT-NP* in vitro*

Based on our previous work, in the presence of 400 U L^-1^ GGT and 2 mM GSH at 37 °C in PBS buffer for 2 h, 200 μM **Glu-DOTA-Gd-CBT** could convert to **DOTA-Gd-CBT-NP**. TEM image showed that the average diameter of** DOTA-Gd-CBT-NP** was 48.2 ± 8.5 nm (Figure 1B and Figure S1) and dynamic light scattering (DLS) measurement indicated that its mean hydrated diameter was 51.9 ± 6.4 nm (Figure 1C). Time-course DLS measurements also indicated that **DOTA-Gd-CBT-NP** was stabile in PBS buffer up to 12 h (Figure S2). These results verified the GGT-guided formation of **DOTA-Gd-CBT-NP*** in vitro*. We firstly measured the r_1_ and r_2_ values of Group “**Glu-DOTA-Gd-CBT** + GGT” (*i.e.*, **DOTA-Gd-CBT-NP**), Group **Glu-DOTA-Gd-CBT**, and Group Gd-DTPA at low magnetic field (1.0 T). In detail, the r_1_ value of Group “**Glu-DOTA-Gd-CBT** + GGT” was measured to be 6.77 mM^-1^ s^-1^, Group **Glu-DOTA-Gd-CBT** be 4.53 mM^-1^ s^-1^, and Group Gd-DTPA be 3.31 mM^-1^ s^-1^. And the r_2_ value of Group “**Glu-DOTA-Gd-CBT** + GGT” was measured to be 6.18 mM^-1^ s^-1^, Group **Glu-DOTA-Gd-CBT** be 4.33 mM^-1^ s^-1^, and Group Gd-DTPA be 5.22 mM^-1^ s^-1^ (Figure 2A and 2B). These results suggested that **DOTA-Gd-CBT-NP** increased the r_1_ value of **Glu-DOTA-Gd-CBT** by 1.49 folds and increased r_2_ value of **Glu-DOTA-Gd-CBT** by 1.43 folds, respectively. The low r_2_/r_1_ ratio 0.91 of **DOTA-Gd-CBT-NP** indicating that it could serve as T_1_ CAs at 1.0 T (Table S1). Then r_1_ and r_2_ values at 9.4 T were calculated. As shown in Figure 2C and 2D, the r_1_ value of Group “**Glu-DOTA-Gd-CBT** + GGT” was measured to be 2.84 mM^-1^ s^-1^, Group **Glu-DOTA-Gd-CBT** be 3.86 mM^-1^ s^-1^, and Group Gd-DTPA be 2.70 mM^-1^ s^-1^, which suggested that **DOTA-Gd-CBT-NP** decreased the r_1_ value of **Glu-DOTA-Gd-CBT** by 0.74 folds. The r_2_ value of Group “**Glu-DOTA-Gd-CBT** + GGT” was measured to be 33.6 mM^-1^ s^-1^, Group **Glu-DOTA-Gd-CBT** be 2.14 mM^-1^ s^-1^, and Group Gd-DTPA be 5.82 mM^-1^ s^-1^, indicating **DOTA-Gd-CBT-NP** dramatically enhanced the r_2_ value of **Glu-DOTA-Gd-CBT** by 15.7 folds. Therefore, the high r_2_/r_1_ ratio 11.8 indicating that **DOTA-Gd-CBT-NP** could act as T_2_ CAs at 9.4 T (Table S1). The *in vitro* study verified that GGT-responsive formation of **DOTA-Gd-CBT-NP** and it could act as T_1_ or T_2_ CAs at 1.0 T or 9.4 T, respectively.

### MR property of DOTA-Gd-CBT-NP in cell

After *in vitro* study, we then applied **Glu-DOTA-Gd-CBT** for MRI of GGT activity in human hepatoma (HepG2) cells. Before that, cytotoxicity was studied. After incubation with 400 μM **Glu-DOTA-Gd-CBT**, 85% of the cells survived up to 8 h, indicating that 200 μM **Glu-DOTA-Gd-CBT** is safe for living cells (Figure S3). HepG2 cells were divided into three groups: cells in Group **Glu-DOTA-Gd-CBT** were incubated with 200 μM **Glu-DOTA-Gd-CBT** for 4 h; cells in Group “DON + **Glu-DOTA-Gd-CBT**” were pretreated with 2 mM 6-diazo-5-oxo-L-norleucine (DON, one type of GGT inhibitors) for 0.5 h then incubated with 200 μM **Glu-DOTA-Gd-CBT** for 4 h; cells in Group Gd-DTPA were incubated with 200 μM Gd-DTPA for 4 h. Inductively coupled plasma-mass spectrometry (ICP-MS) was used to measure the Gd concentrations in HepG2 cells. In detail, Gd concentration in Group **Glu-DOTA-Gd-CBT** (35.6 μM) was higher than that in Group “DON + **Glu-DOTA-Gd-CBT**” (27.9 μM) and Group Gd-DTPA (24.3 μM), which suggested the higher uptake efficiency of **Glu-DOTA-Gd-CBT** (17.8%) than Gd-DTPA (12.2%). As shown in Figure 3A and 3B, the r_1_ value of HepG2 cells in Group **Glu-DOTA-Gd-CBT** was measured to be 6.40 mM^-1^ s^-1^, Group “DON + **Glu-DOTA-Gd-CBT**” be 2.77 mM^-1^ s^-1^, and Group Gd-DTPA be 4.36 mM^-1^ s^-1^ at low magnetic field (1.0 T). And the r_2_ value of Group **Glu-DOTA-Gd-CBT** was measured to be 5.76 mM^-1^ s^-1^, Group “DON + **Glu-DOTA-Gd-CBT**” be 4.39 mM^-1^ s^-1^, and Group Gd-DTPA be 4.64 mM^-1^ s^-1^ at 1.0 T. These results indicated that intracellular formation of **DOTA-Gd-CBT-NP** by GGT increased the r_1_ value of **Glu-DOTA-Gd-CBT** by 2.31 folds and the r_2_ value of **Glu-DOTA-Gd-CBT** by 1.31 folds, respectively. The low r_2_/r_1_ ratio 0.90 of **DOTA-Gd-CBT-NP** indicating that it could serve as T_1_ CAs at 1.0 T (Table S2). The r_1_ value of cells in Group **Glu-DOTA-Gd-CBT** was measured to be 4.11 mM^-1^ s^-1^, Group “DON + **Glu-DOTA-Gd-CBT**” be 3.19 mM^-1^ s^-1^, and Group Gd-DTPA be 2.85 mM^-1^ s^-1^ at high magnetic field (9.4 T) (Figure 3C), which suggested that **DOTA-Gd-CBT-NP** increased the r_1_ value of **Glu-DOTA-Gd-CBT** by 1.29 folds. The r_2_ value of cells in Group **Glu-DOTA-Gd-CBT** was measured to be 32.2 mM^-1^ s^-1^, Group “DON + **Glu-DOTA-Gd-CBT**” be 6.81 mM^-1^ s^-1^, and Group Gd-DTPA be 6.13 mM^-1^ s^-1^ at high magnetic field (9.4 T) (Figure 3D), indicating that **DOTA-Gd-CBT-NP** obviously enhanced the r_2_ value of **Glu-DOTA-Gd-CBT** by 4.73 folds. Therefore, the high r_2_/r_1_ ratio 7.83 suggesting that **DOTA-Gd-CBT-NP** could act as T_2_ CAs at 9.4 T (Table S2). These results confirmed that intracellular GGT-responsive formation of **DOTA-Gd-CBT-NP** could act as T_1_ or T_2_ CAs at 1.0 T or 9.4 T, respectively.

### MR property of DOTA-Gd-CBT-NP* in vivo*

**Figure 4:**
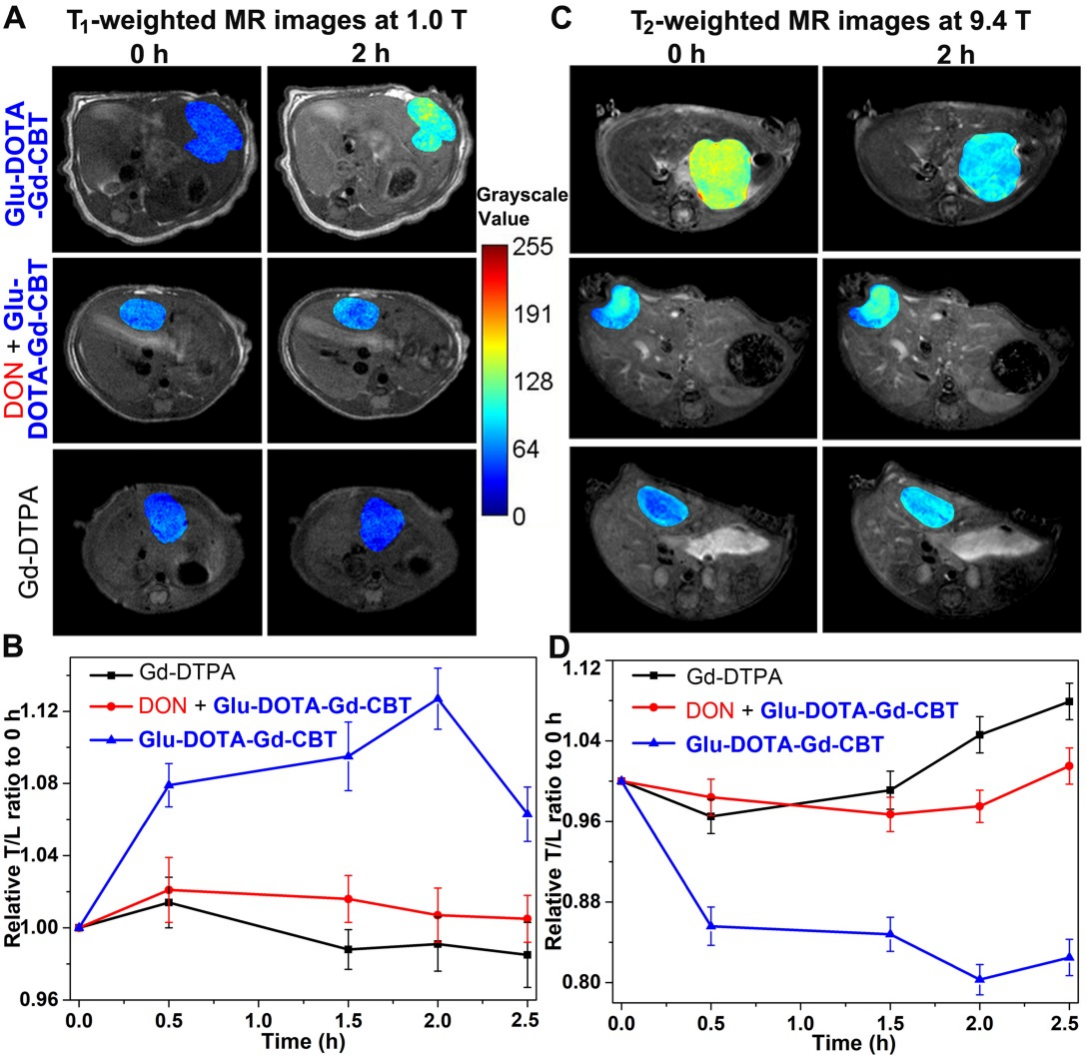
T_1_-weighted transverse MR images at 1.0 T (A) and T_2_-weighted transverse MR images at 9.4 T (C) of liver tumors in Group **Glu-DOTA-Gd-CBT**, Group “DON+**Glu-DOTA-Gd-CBT**” and Group Gd-DTPA at 0 h and 2 h. Normalized time course T/L contrast ratios of T_1_ values in Figure S4 (B) and of T_2_ values in Figure S9 (D).

After cell study, we then investigated T_1_ and T_2_ MRI of liver tumor *in vivo*. Nude mice were transplanted with 1 mm^3^ HepG2 tumor tissue into the liver *in situ*. After the diameter of liver tumors to 1 cm, we tested the GGT activity (313.3 ± 45.3 U L^-1^, Table S3) of liver tumor lysates, which is at a high level. Then mice were randomly divided into three groups: mice in Group **Glu-DOTA-Gd-CBT** were treated with 0.08 mmol kg^-1^ **Glu-DOTA-Gd-CBT** through tail intravenous (i.v.) injection; mice in Group “DON + **Glu-DOTA-Gd-CBT**” were pre-administered with 0.25 mmol kg^-1^ DON for 0.5 h followed by injection of 0.08 mmol kg^-1^ **Glu-DOTA-Gd-CBT**; mice in Group Gd-DTPA received injections of Gd-DTPA at 0.08 mmol kg^-1^. Dynamic T_1_- and T_2_-weighted transverse MR images at low magnetic field (1.0 T) were conducted at first. T_1_ MR contrast of liver tumors in Group **Glu-DOTA-Gd-CBT** reached to its maximum at 2 h while that in Group DON + **Glu-DOTA-Gd-CBT** and Group Gd-DTPA increased slightly at 0.5 h (Figure S4). Typical T_1_ MR images of tumors were shown in Figure 4A. Tumor-to-liver (T/L) contrast ratios of their T_1_ values were displayed in Figure 4B. The T/L ratio of T_1_ values in Group **Glu-DOTA-Gd-CBT** at 2 h was 112.7% of that at 0 h while those in Group “DON + **Glu-DOTA-Gd-CBT**” and Group Gd-DTPA at 0.5 h were 102.1% and 101.4% of theirs at 0 h, respectively. T_2_ MR contrast of liver tumors in three groups showed slight decrease at 0.5 h (the T/M ratios were 96.0% for Group **Glu-DOTA-Gd-CBT**, 98.7% for Group “DON + **Glu-DOTA-Gd-CBT**”, and 98.3% for Group Gd-DTPA) (Figures S5-S6). Then we studied the dynamic T_1_ and T_2_ MR images of liver tumors at high magnetic field (9.4 T). T_1_-weighted MR contrast in three groups slightly enhanced at 0.5 h (the T/M ratios were 104.3% for Group **Glu-DOTA-Gd-CBT**, 102.9% for Group “DON + **Glu-DOTA-Gd-CBT**”, and 106.8% for Group Gd-DTPA to those at 0 h) (Figures S7-S8). As expected, T_2_-weighted MR contrast in Group **Glu-DOTA-Gd-CBT** decreased to its minimum at 2 h (the T/M ratio was 80.3% to that at 0 h). In contrast, neither Group “DON + **Glu-DOTA-Gd-CBT**” nor Group Gd-DTPA showed obvious T_2_ contrast change (the lowest T/M ratios were 98.4% and 96.5% to those at 0 h for Group “DON + **Glu-DOTA-Gd-CBT**” and Group Gd-DTPA at 0.5 h, respectively) (Figures 4C-4D, S9). These results indicated the GGT-instructed formation of **DOTA-Gd-CBT-NP** from **Glu-DOTA-Gd-CBT** could specifically increase the T_1_ MR imaging of liver tumor at 1.0 T and largely enhance T_2_ MR imaging of liver tumor at 9.4 T. After 2 h, T/L contrast ratio started to decrease which was consistent to our previous study 24. At last, we measured gadolinium concentrations in liver tumors and other organs (hearts, livers, spleens, lungs, and kidneys) of these mice after MRI at 2.5 h. In addition to the organs involved in metabolism (livers and kidneys), tumors have the highest concentration of Gd (Figure S10, Table S4). Specifically, the average Gd content of tumors in Group **Glu-DOTA-Gd-CBT** (5.35 μg/g) was much higher than that in Group “DON + **Glu-DOTA-Gd-CBT**” (1.91 μg/g) or Group Gd-DTPA (1.22 μg/g). This result further indicated that **DOTA-Gd-CBT-NP** extended the retention time of Gd in liver tumors and thereafter largely enhanced the MRI contrast of liver tumors.

## Conclusions

In summary, we integrally studied the MR property of GGT-guided formation of Gd-nanoparticle for liver tumor imaging at both low and high magnetic fields. We found that **DOTA-Gd-CBT-NP** possesses low r_2_/r_1_ ratio 0.91 at 1.0 T which specifically enhanced T_1_ MR imaging of liver tumors (the T/L ratio of was 112.7%) at low magnetic field, and high r_2_/r_1_ ratio 11.8 at 9.4 T which largely enhanced T_2_ MR imaging of liver tumors (the T/M ratio was 80.3%) at high magnetic field. Therefore, we expect that our GGT-instructed Gd-nanoparticle formation could be applied for MRI diagnosis of early liver cancer in clinic at low or high magnetic field when the 9.4 T MR machine is clinically available in the future.

## Supplementary Material

Supplementary figures and tables.
