# Supplementary material for: Simultaneous enhancement of T1 and T2 magnetic resonance imaging of liver tumor at respective low and high magnetic fields
Source: Theranostics. 2022 Jan 1;12(1):410–7. doi: 10.7150/thno.67155 (PMC8690926; doi:10.7150/thno.67155)
Supplement: Supplementary file 1 — Supplementary figures and tables. [file thnov12p0410s1.pdf]

Supplementary Materials

Simultaneous enhancement of T_1 and T_2 magnetic resonance imaging of liver tumor at respective low and high magnetic fields

Huan Li,[#1] Zijuan Hai,[#2] Liwei Zou,[1] Lele Zhang,[2] Lulu Wang,[4] Longsheng Wang[*1] and Gaolin Liang[*3]

1. Department of Radiology, the Second Hospital of Anhui Medical University, Hefei, Anhui 230601, China
2. Key Laboratory of Structure and Functional Regulation of Hybrid Materials, Ministry of Education, Institutes of Physical Science and Information Technology, Anhui University, Hefei, Anhui 230601, China
3. State Key Laboratory of Bioelectronics, School of Biological Science and Medical Engineering, Southeast University, Nanjing, Jiangsu 210096, China
4. High Magnetic Field Laboratory, Hefei Institutes of Physical Science, Chinese Academy of Sciences, Hefei, Anhui 230031, China

[#]These authors contributed equally to this work.

E-mail: wanglongsheng@ahmu.edu.cn (L.-S. W.), gliang@seu.edu.cn (G.-L. L.).

Contents:

1. Supplementary figures and tables

1. Supplementary figures and tables

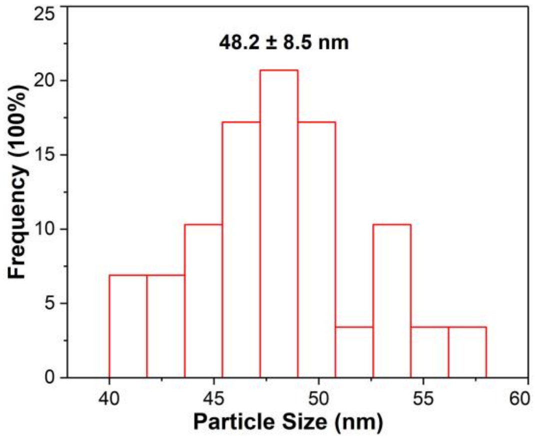

Figure S1. Statistics of size distribution of **DOTA-Gd-CBT-NP** in Figure 1B.

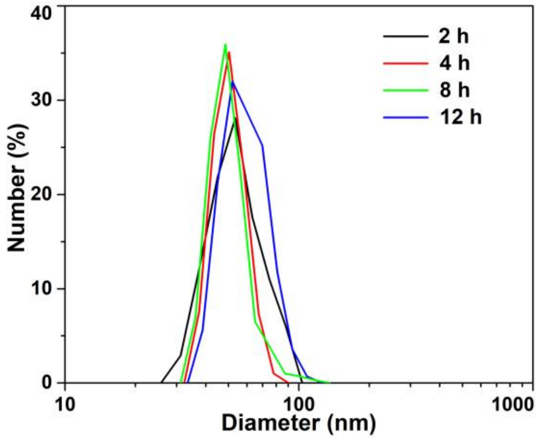

Figure S2. Time-course DLS measurement of **DOTA-Gd-CBT-NP** in PBS buffer.

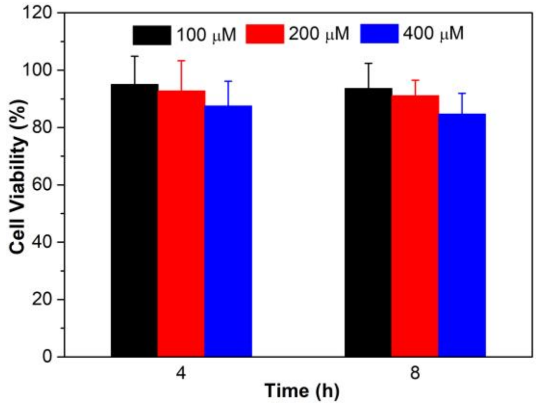

Figure S3. Cell viability of HepG2 cells incubated with **Glu-DOTA-Gd-CBT** at different concentrations for 4 h, and 8 h. Each error bar represents the standard deviation of three independent experiments.

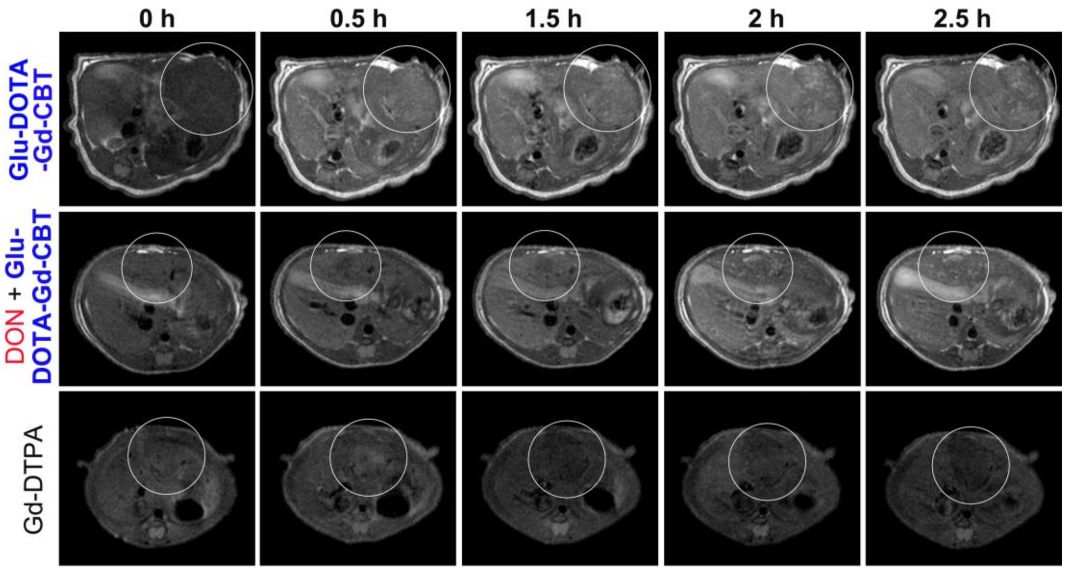

Figure S4. *In vivo* dynamic T_1-weighted transverse MR images of **Glu-DOTA-Gd-CBT**-injected mice (top row), DON-pretreated and then **Glu-DOTA-Gd-CBT**-injected mice (middle row), and Gd-DTPA-injected mice (bottom row) at low magnetic field (1.0 T). White circles indicate the liver tumors.

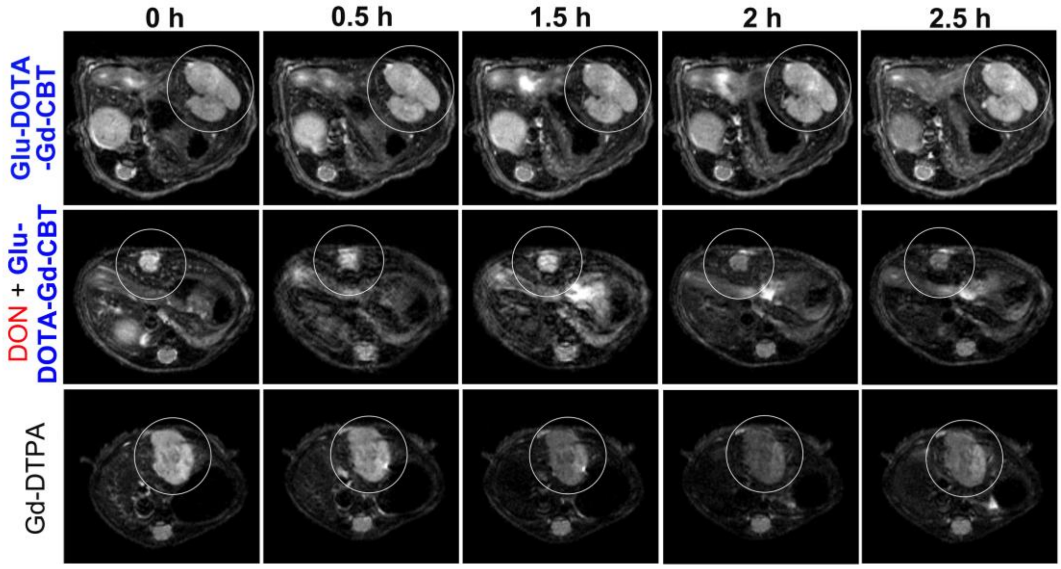

Figure S5. *In vivo* dynamic T_2-weighted transverse MR images of **Glu-DOTA-Gd-CBT**-injected mice (top row), DON-pretreated and then **Glu-DOTA-Gd-CBT**-injected mice (middle row), and Gd-DTPA-injected mice (bottom row) at low magnetic field (1.0 T). White circles indicate the liver tumors.

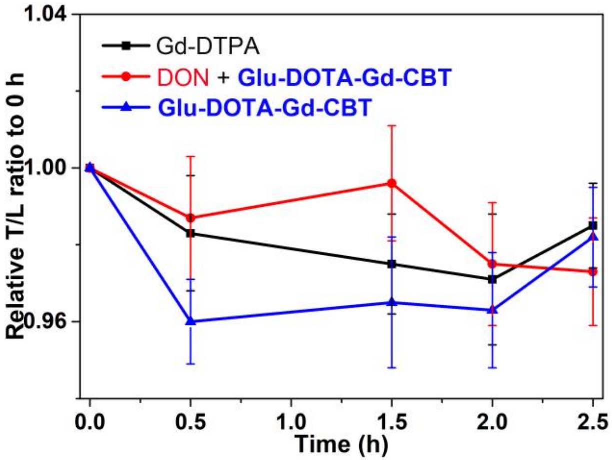

Figure S6. Normalized time course relative tumor-to-liver (T/L) contrast ratios of T_2 values at low magnetic field (1.0 T) in Figure S5. Each error bar represents the standard deviation of three independent experiments.

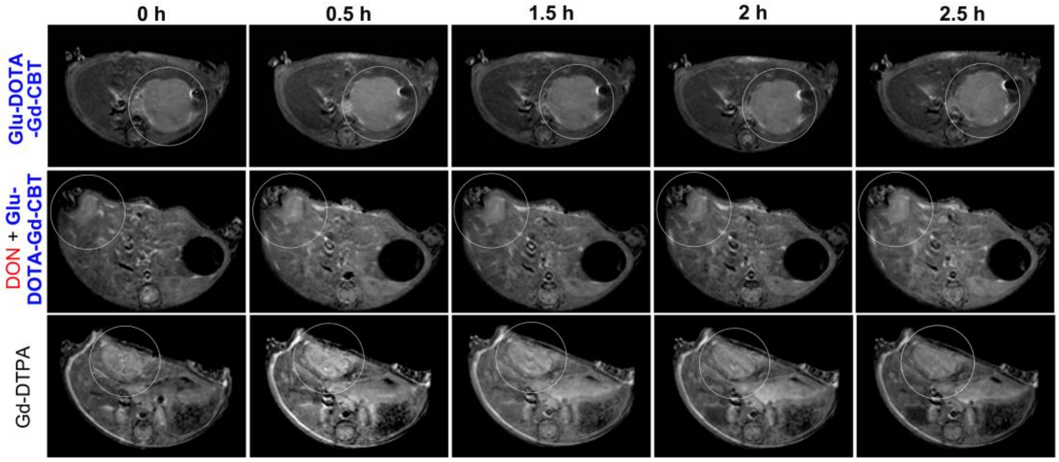

Figure S7. *In vivo* dynamic T_1-weighted transverse MR images of **Glu-DOTA-Gd-CBT**-injected mice (top row), DON-pretreated and then **Glu-DOTA-Gd-CBT**-injected mice (middle row), and Gd-DTPA-injected mice (bottom row) at high magnetic field (9.4 T). White circles indicate the liver tumors.

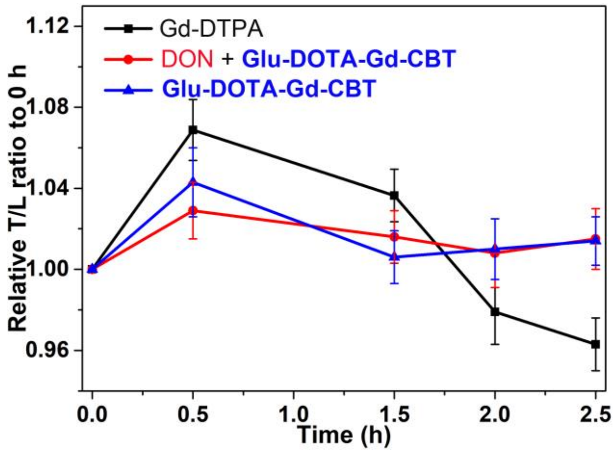

Figure S8. Normalized time course relative tumor-to-liver (T/L) contrast ratios of T_1 values at high magnetic field (9.4 T) in Figure S7. Each error bar represents the standard deviation of three independent experiments.

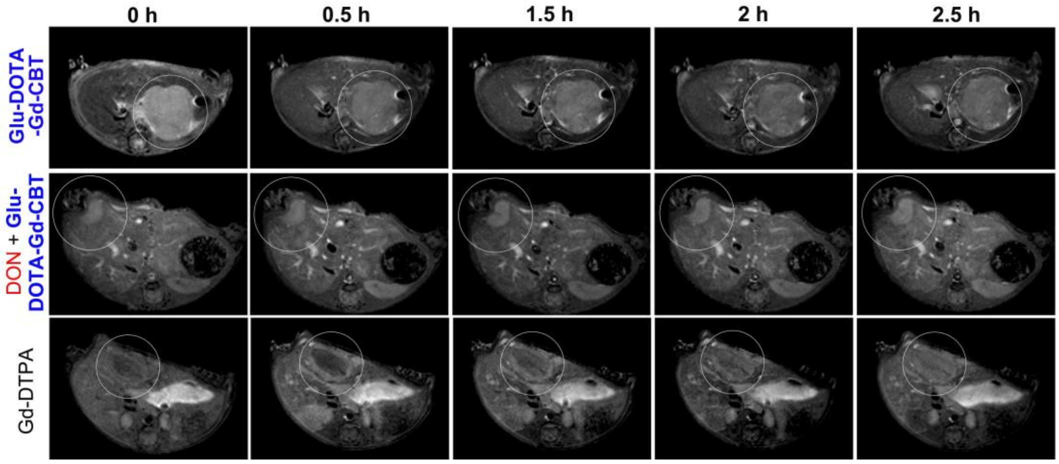

Figure S9. *In vivo* dynamic T_2-weighted transverse MR images of **Glu-DOTA-Gd-CBT**-injected mice (top row), DON-pretreated and then **Glu-DOTA-Gd-CBT**-injected mice (middle row), and Gd-DTPA-injected mice (bottom row) at high magnetic field (9.4 T). White circles indicate the liver tumors.

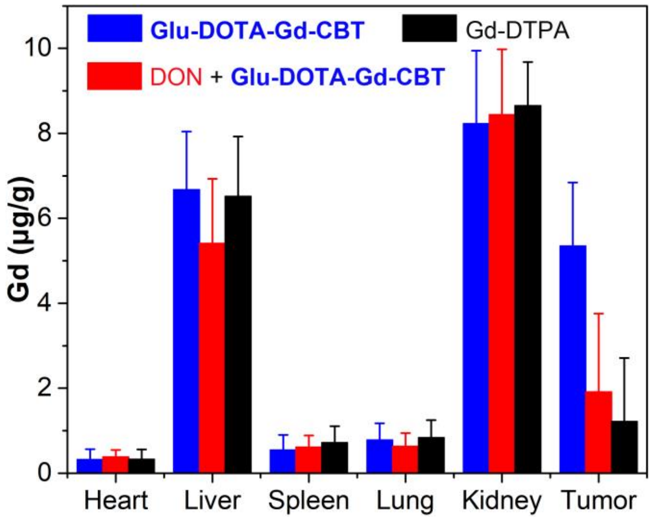

Figure S10. The contents of Gd (μg/g, determined with ICP-MS) in tumors and organs of three groups after MRI at 2.5 h.

Table S1. The r_2/r_1 ratio values of **DOTA-Gd-CBT-NP**, **Glu-DOTA-Gd-CBT** and Gd-DTPA at low (1.0 T) and high (9.4 T) magnetic field.

	r_2/r_1 ratio value at 1.0 T	r_2/r_1 ratio value at 9.4 T
**DOTA-Gd-CBT-NP**	0.91	11.8
**Glu-DOTA-Gd-CBT**	0.96	0.55
Gd-DTPA	1.58	2.16

Table S2. The r_2/r_1 ratio values of cells in Group **Glu-DOTA-Gd-CBT**, Group "DON + **Glu-DOTA-Gd-CBT**", and Group Gd-DTPA at low (1.0 T) and high (9.4 T) magnetic field.

	r_2/r_1 ratio value at 1.0 T	r_2/r_1 ratio value at 9.4 T
**Glu-DOTA-Gd-CBT**	0.90	7.83
DON + **Glu-DOTA-Gd-CBT**	1.58	2.13
Gd-DTPA	1.06	2.15

Table S3. GGT activity in liver tumor lysates.

Diameter of Tumor Size (mm)	Increased O.D. Value at 405 nm between 1 min	GGT activity (U/L)
10	0.037 ± 0.015	313.3 ± 45.3

Table S4. The contents of Gd (μg/g, determined with ICP-MS) in tumors and main organs of three groups after MRI at 2.5 h.

	Heart	Liver	Spleen	Lung	Kidney	Tumor
**Glu-DOTA-Gd-CBT**	0.32 ± 0.24	6.67 ± 1.37	0.55 ± 0.35	0.78 ± 0.39	8.23 ± 1.78	5.35 ± 1.37
DON+**Glu-DOTA-Gd-CBT**	0.39 ± 0.16	5.41 ± 1.52	0.62 ± 0.27	0.63 ± 0.31	8.44 ± 1.63	1.91 ± 1.65
Gd-DTPA	0.33 ± 0.23	6.52 ± 1.41	0.72 ± 0.39	0.84 ± 0.41	8.65 ± 1.24	1.22 ± 1.03
